# Islas interdependientes: políticas sanitarias y prácticas médicas en la asistencia, gestión e investigación de una enfermedad desatendida en el Sur Global

**DOI:** 10.1590/0102-311XES114225

**Published:** 2026-01-09

**Authors:** Laurencia Silveti, Adelyne Maria Mendes Pereira

**Affiliations:** 1 Instituto de Estudios para el Desarrollo Social, Universidad Nacional de Santiago del Estero/Consejo Nacional de Investigaciones Científicas y Técnicas, Santiago del Estero, Argentina.; 2 Escola Nacional de Saúde Pública Sergio Arouca, Fundação Oswaldo Cruz, Rio de Janeiro, Brasil.

**Keywords:** Práctica Institucional, Enfermedades Desatendidas, Salud Global, Política de Salud, Institutional Practice, Neglected Diseases, Global Health, Health Policy, Prática Institucional, Doenças Negligenciadas, Saúde Global, Política de Saúde

## Abstract

Esta investigación analiza prácticas médicas vinculadas con la hidatidosis en la provincia de Santiago del Estero, Argentina. Esta zoonosis parasitaria e infecciosa es reconocida como un importante problema de salud pública en América del Sur. Sin embargo, forma parte del conjunto de enfermedades clasificadas como desatendidas por la Organización Mundial de la Salud, que afectan principalmente a poblaciones pobres que habitan zonas rurales, remotas o marginales, con acceso limitado a los servicios de salud. El objetivo del estudio es analizar la configuración de la hidatidosis como problema sanitario, a partir del abordaje de las prácticas médicas implicadas en su atención, gestión e investigación, desde perspectivas del Sur Global. Para ello, se adoptó un diseño cualitativo que incluyó entrevistas a profesionales de la salud con experiencia en el tema y la construcción de un corpus documental compuesto por informes técnicos (2015-2023) y políticas públicas en distintos niveles. El estudio identifica tres características principales de las prácticas en torno a la hidatidosis en Santiago del Estero: (1) una gestión médica de corte colonial y autoritario, en tensión con la autonomía técnica local debilitada por la falta de apoyo político; (2) trabajo y atención fragmentados, con escasa articulación intersectorial y predominio de modelos biomédicos y tecnocráticos; y (3) una investigación en salud alineada con prioridades globales, poco vinculada a necesidades locales. Estas dinámicas expresan una dependencia estructural de enfoques estandarizados, en detrimento de estrategias contextualizadas e integrales de salud pública.

## Abstract (en)

This study analyzes medical practices related to hydatidosis in the province of Santiago del Estero, Argentina. This parasitic zoonotic disease is recognized as a significant public health problem in South America. Nevertheless, it is included among the neglected diseases as classified by the World Health Organization - those that primarily affect the poorest populations living in rural, remote, or marginalized areas with limited access to health services. The objective is to examine how hydatidosis is configured as a health issue through an analysis of medical practices involved in its care, management, and research, from Global South perspectives. A qualitative design was employed, including interviews with healthcare professionals working in the field, as well as the construction of a documentary corpus comprising technical reports from 2015 to 2023 and public policies at different levels. The study identifies three main characteristics of practices related to hydatidosis in Santiago del Estero: (1) a medical management style marked by colonial and authoritarian traits, in tension with local technical autonomy and weakened by a lack of political support; (2) fragmented healthcare processes, with limited intersectoral coordination and a predominance of biomedical and technocratic models; and (3) health research aligned with global priorities, often disconnected from local needs. These dynamics reflect a structural dependence on standardized approaches, to the detriment of contextualized and comprehensive public health strategies.

## Abstract (pt)

Esta pesquisa analisa as práticas médicas vinculadas à hidatidose na província de Santiago del Estero, Argentina. Essa zoonose parasitária e infecciosa é reconhecida como um importante problema de saúde pública na América do Sul. No entanto, faz parte do grupo de doenças classificadas como negligenciadas pela Organização Mundial da Saúde, que afetam principalmente populações pobres que vivem em áreas rurais, remotas ou marginais com acesso limitado a serviços de saúde. O objetivo do estudo é analisar a configuração da hidatidose como um problema de saúde, a partir das práticas médicas envolvidas em seu cuidado, manejo e pesquisa, a partir das perspectivas do Sul Global. Para tanto, adotou-se um delineamento qualitativo que incluiu entrevistas com profissionais de saúde com experiência no tema e a construção de um *corpus* documental composto por relatórios técnicos (2015-2023) e políticas públicas em diferentes níveis. O estudo identifica três características principais das práticas de hidatidose em Santiago del Estero: (1) gestão médica colonial e autoritária, em tensão com a autonomia técnica local fragilizada pela falta de apoio político; (2) trabalho e cuidado fragmentados, com pouca coordenação intersetorial e predomínio de modelos biomédicos e tecnocráticos; e (3) pesquisa em saúde alinhada às prioridades globais e pouco conectada às necessidades locais. Essas dinâmicas expressam uma dependência estrutural de abordagens padronizadas, em detrimento de estratégias de saúde pública contextualizadas e abrangentes.

## Introducción

Las enfermedades catalogadas como desatendidas -también denominadas “tropicales”- son un grupo de enfermedades infecciosas relacionadas con la pobreza y la precariedad ambiental y de vivienda, que afectan principalmente a poblaciones vulneradas ^1^. Dentro de estas enfermedades, se encuentra la hidatidosis o equinococosis quística, de la que se ocupa este trabajo. Esta zoonosis, causada por la forma larvaria de *Echinococcus granulosus*, tiene un ciclo de transmisión que involucra principalmente a perros como hospedadores definitivos y a herbívoros (por ejemplo, ovejas, cabras, etc.) como intermediarios. La infección humana se produce accidentalmente por ingestión de huevos parasitarios presentes en alimentos, agua o suelo contaminados, o bien, por contacto con perros parasitados, generando quistes viscerales que pueden permanecer asintomáticos hasta alcanzar gran tamaño o romperse. Su diagnóstico combina clínica, imágenes y serología, y el tratamiento varía según el tipo de quiste, puede ser medicamentoso, quirúrgico o percutáneo, dependiendo de las características del quiste y del paciente.

Más allá del abordaje biomédico-medicalizado, aunque la transmisión podría evitarse mediante medidas concretas -como desparasitar periódicamente a los perros, evitar alimentarlos con vísceras crudas, entre otras-, la persistencia de la enfermedad se vincula con determinantes sociales que sostienen su transmisión endémica: precariedad rural, déficit de acceso a control veterinario, a salud pública y a servicios básicos, y desigualdades vinculadas al legado histórico colonial que marginaliza poblaciones y territorios periféricos. Como enfermedad desatendida, su evaluación epidemiológica se ve obstaculizada por datos incompletos, dispersos y metodológicamente heterogéneos, lo que refuerza la necesidad de sistemas unificados y accesibles de recolección y análisis de información ^2^. En Argentina, Brasil, Chile, Perú y Uruguay se diagnostican anualmente alrededor de 5.000 nuevos casos de hidatidosis ^3^. En Argentina predomina la equinococosis quística, mientras que en Brasil coexiste con la neotropical ^4^.

Los abordajes estatales regionales formales comenzaron en 1941, cuando se creó la Asociación Internacional de Hidatidología, conformada por Argentina, Brasil y Uruguay. Pese a la existencia de políticas de control desde mediados del siglo XX ^5^ ^,^ ^6^ ^,^ ^7^ -incluyendo el Proyecto Subregional Cono Sur impulsado por la Organización Panamericana de la Salud (OPS)/Organización Mundial de la Salud (OMS)- la enfermedad persiste en poblaciones rurales empobrecidas, con bajo interés para el complejo médico-industrial. Sus repercusiones incluyen mortalidad, discapacidad y efectos económicos y sociales que refuerzan ciclos de exclusión y estigmatización histórica.

El sostenimiento de la desatención tensiona la efectividad de las políticas públicas, fundamentalmente aquellas del paradigma de Salud Global. La Salud Global emergió como una respuesta transnacional a desafíos sanitarios contemporáneos, caracterizada por la creciente interdependencia entre países en relación con la salud de sus poblaciones ^8^. Sin embargo, la Salud Global ha sido criticada por reproducir enfoques tecnocráticos y biomédicos que invisibilizan las condiciones estructurales que producen enfermedad, al tiempo que refuerzan relaciones de colonialidad entre el Norte y el Sur Global ^9^. Estas relaciones coloniales se vinculan con la delimitación territorial de enfermedades denominadas “tropicales desatendidas”: la construcción de un nombre basada en términos geográficos coincide con la delimitación de los espacios coloniales, territorios vivos de pueblos, culturas, saberes, políticas, conocimientos y situaciones sanitarias sometidas al extractivismo ^7^.

En el marco de la Salud Global, la hidatidosis ocupa un lugar marginal en términos de inversión en investigación y desarrollo. Su baja letalidad, larga evolución clínica y concentración en áreas periféricas contribuyen a su invisibilización en las agendas sanitarias dominantes, lo que condiciona las posibilidades terapéuticas, subraya la fragmentación en los sistemas de atención y reproduce dinámicas de exclusión ^10^ ^,^ ^11^.

Además, desde las ciencias sociales se destacan tensiones entre conocimientos técnicos y saberes locales que reflejan desigualdades epistémicas e inciden en procesos de diagnóstico y tratamiento, así como en la legitimidad percibida de las políticas sanitarias ^12^. A su vez, la fragmentación de respuestas sectoriales obstaculiza la implementación efectiva de estrategias sostenidas de control y prevención en este tipo de enfermedades ^13^ ^,^ ^14^.

Por su parte, las prácticas médicas sobre hidatidosis constituyen procesos situados que condensan relaciones de poder, trayectorias institucionales/profesionales, experiencias y sentidos sobre la enfermedad que exceden lo técnico. De ello, el análisis de las prácticas médicas, entendidas como un conjunto de acciones orientadas a diagnosticar, investigar, tratar y mejorar la salud en un marco político-institucional determinado ^15^, se torna relevante.

Estas prácticas médicas sobre la hidatidosis se desarrollan en un determinado sistema de salud. Un sistema de salud se puede definir como un conjunto de relaciones políticas, económicas e institucionales que conducen los procesos relacionados con la salud de determinada población y se materializan en organizaciones, normativas y servicios orientados a la producción de resultados coherentes con el concepto de salud dominante ^16^. Su configuración deviene de las capacidades de cada contexto geopolítico.

Esta investigación se apoya en el argumento de que una mirada desde las ciencias sociales permite identificar que estas prácticas se desarrollan en un entramado de relaciones entre saberes biomédicos, condiciones de vida y dinámicas institucionales que moldean los modos de atención, gestión e investigación. Si bien el abordaje biomédico de la hidatidosis ha avanzado en términos clínico-quirúrgicos y parasitológicos, la intervención sanitaria continúa atravesada por limitaciones estructurales, discontinuidades institucionales y saberes en disputa ^17^. Partimos, entonces, del supuesto de que existen dinámicas institucionales atravesadas por la colonialidad que afectan el accionar de los equipos y reproducen la desatención.

Entendiendo a la enfermedad como proceso socioepidemiológico complejo ^18^ y reconociendo los modos en que estas toman relevancia para constituirse como problemas públicos ^19^, la pregunta que orienta este estudio es ¿de qué modo se configura la hidatidosis como enfermedad desatendida en las prácticas médicas de atención, gestión e investigación en contextos específicos del Sur Global?

En síntesis, el objetivo de este trabajo es analizar políticas sanitarias y prácticas médicas en la asistencia, gestión e investigación de la hidatidosis como enfermedad infecciosa desatendida en contextos específicos del Sur Global, en este caso, la provincia de Santiago del Estero, Argentina, entre 2015 y 2023. Para situar el trabajo, cabe advertir que Argentina es un país federal que presenta elevada desigualdad en términos de empleo, protección social, acceso a salud y educación, calidad de vivienda y hábitat ^20^. El sistema de salud sostiene una estructura descentralizada con tres subsistemas: público, de seguridad social (obras sociales) y privado. Se observa una gran heterogeneidad y desigualdad en el acceso a servicios y personal de salud entre provincias y regiones ^21^.

## Metodología

**Figura 1:**
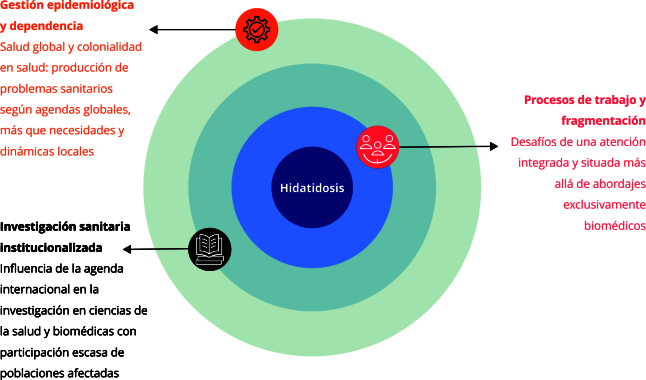
Matriz conceptual de análisis.

Desde un diseño cualitativo interpretativo-crítico, se focalizó en la sistematización y análisis de prácticas médicas en hidatidosis en una provincia del noroeste argentino. A partir de referentes teóricos que abordan las implicaciones de la colonialidad sobre las políticas y prácticas médicas en enfermedades infecciosas desatendidas en el contexto del Sur Global ^7^ ^,^ ^16^ ^,^ ^22^ ^,^ ^23^ ^,^ ^24^, fue construida una matriz de análisis (Figura 1) formada por tres dimensiones: (1) gestión epidemiológica y dependencia, (2) procesos de trabajo-atención y fragmentación y (3) investigación sanitaria institucionalizada, alcances y limitaciones.

Fuente: elaboración propia a partir de Oliveira ^7^, Lobato & Giovanella ^16^, Gonçalves et al. ^22^, Pereira et al. ^23^ y Carrillo et al. ^24^.

Se realizó un estudio de caso en Santiago del Estero entre 2015 -cuando la provincia empezó a producir actividades y registros sistemáticos sobre la hidatidosis- y 2023, momento en que se identifica una disminución de acciones institucionales organizadas sobre esta enfermedad. La justificación para el estudio de caso está en la importancia de esta enfermedad para la región noroeste, siendo Santiago del Estero la segunda provincia con más número de casos en 2023 (29 casos confirmados) ^25^.

Respecto del sistema de salud de Santiago del Estero, este sigue la organización sanitaria nacional mencionada anteriormente. El subsector público se organiza en tres niveles de atención según tipo de necesidad y capacidad de respuesta, con centros de atención distribuidos en toda la provincia ^26^. El enfoque sanitario provincial se vincula con *Una Salud*, perspectiva internacional e integral de abordaje unificado para equilibrar y optimizar la salud de las personas, los animales y los ecosistemas, entendiendo sus interdependencias ^13^ ^,^ ^27^.

La selección de Santiago del Estero como escenario no es casual: se trata de una provincia históricamente relegada en términos de infraestructura sanitaria, donde persisten formas de organización de la atención que ponen en evidencia los límites del modelo biomédico hegemónico y la necesidad de considerar la determinación social de la salud. Así, el estudio de caso no solo proporciona bases empíricas, sino que también contribuye a debates más amplios sobre la implementación de políticas sanitarias.

Las estrategias de investigación combinaron seis entrevistas -realizadas entre 2020 y 2023- y análisis documental de todos los informes técnicos producidos (Cuadro 1) en torno a las actividades realizadas entre 2015 y 2023 por la Secretaría Técnica Epidemiológica (STE) del Ministerio de Salud Provincial. Los criterios para selección de participantes fueron que sean trabajadores sanitarios en ejercicio de diferentes cargos de gestión, atención e investigación en relación con la hidatidosis en el sistema de salud provincial al momento de las entrevistas. Estas entrevistas fueron anonimizadas y sus nombres fueron cambiados por letras de la A a la F.

**Cuadro 1:** Descripción de informes técnicos sobre hidatidosis en la provincia de Santiago del Estero, Argentina.

NOMBRE	CONTENIDO	FECHA
Plan Provincial de Hidatidosis. Informe de actividades realizadas durante el periodo 2016-2017	Resumen de actividades desarrolladas entre agosto de 2016 y noviembre de 2017. Definiciones conceptuales, datos epidemiológicos, objetivos del plan provincial de hidatidosis, actividades de campo realizadas, saberes y actores involucrados/as y desafíos pendientes	No establece
Implementación de un Plan Integral de Control de Hidatidosis. Los Telares, Departamento Salavina, Santiago del Estero	Presentación en jornada académica internacional derivada de la propuesta de Plan provincial de hidatidosis antes mencionado	Octubre de 2017
Informe Loreto	Descripción de acciones de control, actores y programas intervinientes y de charlas informativas realizadas (incluye fotografías), con programación de nueva visita (21 de junio)	7 de junio de 2017
Informe Loreto	Descripción de acciones de control, actores y programas intervinientes y de charlas informativas realizadas (incluye fotografías)	21 de junio de 2017
Informe Loreto	Descripción de acciones de control, actores y programas intervinientes y de charlas informativas realizadas (incluye fotografías)	12 de junio y 25 de junio de 2019
Informe Loreto	Descripción de acciones de control, actores y programas intervinientes y de charlas informativas realizadas (incluye fotografías)	23, 24 de septiembre y 1 de octubre de 2019

Fuente: elaboración propia en base al material empírico documental.

De acuerdo con la matriz conceptual de análisis (Figura 1), la codificación abierta del material empírico se desarrolló en tres etapas: (1) lectura y selección de fragmentos representativos según dimensiones y categorías de análisis con aplicación de códigos iniciales para identificar patrones, acciones o significados relevantes; (2) reorganización y comparación de códigos para formar categorías más abstractas; y (3) integración de esas categorías en nociones conceptuales más amplias ^28^. Los resultados son presentados según las tres dimensiones de análisis.

En cumplimiento de los principios éticos de la Declaración de Helsinki (2008) y de la Asociación Médica Mundial, se utilizó consentimiento informado, garantizando confidencialidad y anonimato a las personas participantes. El estudio fue autorizado por autoridades de las instituciones involucradas en reunión del 5 de agosto de 2021.

## Resultados y discusión

Este trabajo analiza políticas sanitarias y prácticas médicas en la asistencia, gestión e investigación de la hidatidosis como enfermedades infecciosas desatendidas en el Sur Global entre 2015 y 2023. Los resultados alcanzados son presentados según las dimensiones de análisis: (1) gestión epidemiológica y dependencia; (2) procesos de trabajo-atención y fragmentación; y (3) investigación sanitaria institucionalizada.

### Gestión epidemiológica y dependencia

El análisis del material empírico acerca de esta dimensión destacó la dependencia y colonialismo interno como importantes categorías para la comprensión de las dinámicas coloniales sobre la gestión epidemiológica de la hidatidosis en el caso de estudio.

De acuerdo con las entrevistas, en el ámbito sanitario provincial se empezó a hablar más sistemáticamente acerca de la hidatidosis en 2015, debido a un plan operativo introducido por la OPS a nivel mundial. Así lo refiere la persona entrevistada A:

“*Fue a partir de esa reunión de OPS por el plan operativo.* (...) *una especie de movida a nivel mundial, que ha coincidido con el aumento de casos detectados.* (...) *Ya se venía trabajando con hidatidosis, mal que mal, porque es una patología de notificación obligatoria, o sea, de tratar de cargarla en el sistema, para que se fundamente un poco las estadísticas y los recursos para trabajar. Pero desde ese momento* [con la OPS] *se empezó a trabajar con profundidad, por un tema de recursos*”.

Previo a este evento, se trabajaba con lineamientos de la OMS registrando casos para recibir medicación, las tareas de detección-prevención no existían, así lo refiere la persona entrevistada B:

“*La cuestión de búsqueda* [de casos de hidatidosis] *no existía. Eso empieza en 2015, con la OPS. Nos contactamos con el Hospital de los Telares y a partir de ahí con las escuelas.* (...) *Porque esas son las pautas, hacer el tamizaje de esta población*”.

Entonces, a partir del desembarco de organismos internacionales como la OPS, se reorganizó y amplió el trabajo sobre hidatidosis y se concentró en el Programa de Zoonosis de la STE que dependía de la Dirección General de Medicina Preventiva. En 2016, con capacitación de la OPS, se conformó -sin resultar formalizado institucionalmente- un equipo interdisciplinario para la detección de hidatidosis quística en escuelas rurales (Los Telares), integrado por médicas/os, veterinarias/os y educadoras/es de salud. Es notable que las personas entrevistadas refieren que, sin la intervención de la OPS, no se hubiera avanzado en generar estrategias de prevención, como la detección precoz. Aunque la experiencia articuló asistencia, gestión e investigación -algo poco habitual-, sus acciones respondieron a lineamientos internacionales más que a aspectos locales, regionales o nacionales específicos. Esto puede estar relacionado a dinámicas de dependencia estructural, donde el Sur Global adopta políticas diseñadas en el Norte con escasa adaptación contextual y la menor resistencia posible ^29^.

Las directrices de OPS/OMS sobre hidatidosis -incluidas en la Agenda Regional 2020-2029- establecen estrategias definidas globalmente con fines de vigilancia epidemiológica, desparasitación veterinaria, diagnóstico clínico, indicadores estándar y esquemas de monitoreo-evaluación técnica ^30^. Estas guías promueven modelos técnicos uniformes, diseñados desde el Norte Global. Aunque, a veces, incluyen lo que denominan “tradicional” lo hacen de forma subalternizada respecto de la biomedicina. De este modo, las guías se presentan como universalmente aplicables, pero rara vez incorporan participación local. Esto opera generando dependencia institucional y epistemológica: poblaciones locales quedan sujetas a instrumentos ajenos que definen prioridades sin su participación, bajo paradigmas verticales importados, como ocurrió con las campañas contra tuberculosis, que reprodujeron lógicas de control técnico y subordinación sanitaria ^31^.

En las entrevistas, se reflejan racionalidades que orientan el funcionamiento de políticas y prácticas médicas desde configuraciones de subalternidad y dependencia. Allí se advierte que los lineamientos de la OPS/OMS sobre hidatidosis no solo articulan un enfoque biomédico centrado en indicadores y protocolos técnicos para el “tamizaje de la población”, sino que también reproducen la colonialidad del conocimiento, al priorizar lógicas biomédicas universales frente a experiencias locales y al construir dependencia técnica. La persona entrevistada C destaca que los protocolos de atención suelen ser rígidos, exigiendo que las personas que atienden y las poblaciones se adapten a ellas: “*ellos* [las familias a las que atienden] *son flexibles, por ahí no es flexible el sistema*”. En el mismo sentido, la entrevistada B refirió: “*Entonces me iba armando e iba viendo de acuerdo a lo que se quería que yo hiciera*”.

Este diseño institucional tiende a reforzar inequidades estructurales y mantener vivas dinámicas de poder tradicionales en la gestión epidemiológica desde la dependencia. En el caso analizado, los lineamientos de organismos internacionales se reconocen como necesarios y oportunos, aunque años después de haber llevado a cabo su implementación, también entraron en tensión con lo que los equipos locales consideraron adecuado, como trabajar con lugares y poblaciones con casos positivos en lugar de un abordaje genérico. De este modo, los países endémicos se convierten en ejecutores de programas diseñados en el Norte, con espacios limitados para autonomía epistémica o intervención comunitaria. Esto se refleja, por ejemplo, a partir de una inquietud emergente respecto de los lineamientos donde la persona entrevistada A refirió:

“*Haber tomado a las escuelas para hacer seguir el movimiento que propone la OPS hace bien y abarca a los niños en edad escolar: detección temprana, claro.* (...) *Pero trabajar miles de miles de horas, ir a escuelas y que no haya nada...* (...) *por ahí uno de la parte técnica quiere hacer muchísimas cosas. Pero va a ser dentro de lo que uno pueda hacer, es decir, las limitaciones.* (...) *Ahí habíamos evaluado y priorizado trabajar con las familias específicamente de los casos positivos. Así que ese sería nuestro próximo paso*”.

El equipo técnico funcionó de 2016 a 2019, cuando la renuncia de su coordinadora por presiones jerárquicas generó descoordinación y hostilidad laboral. La pandemia en 2020 interrumpió sus actividades y, pese a intentos de reactivación, las tensiones internas obstaculizaron la gestión de recursos y decisiones, por ejemplo, al impedir que el equipo gestione la medicación, evidenciando disputas de poder en la implementación del programa. Así lo relata la persona entrevistada A:

“*Nunca han colaborado en el proceso* [dirección de área]*.* (...) *Son cuestiones políticas en las que no entramos.* (...) *Yo reconozco la autoridad, pero voy a hacer lo que es bueno para la salud pública, no para los intereses personales*”.

Estas experiencias reflejan que, en este caso, las decisiones tomadas desde las direcciones de servicios -puesto ocupado principalmente por varones que no trabajan con las comunidades-, se tornaron autoritarias, informales y sin argumentos institucionales claros, situaciones que enfrentaron los equipos técnicos que sí trabajaban en los territorios. Así, se desplazó personal capacitado por motivos personales y se identificaron usos de recursos de forma discrecional, de acuerdo a afinidades y obsecuencias más que a capacidades. Eso afectó a los equipos porque, como refiere la persona entrevistada D:

“*Trabajamos mucho* (...) *y ver que está armado y que lo desarman, nos parte el alma* (...) *y lo hacemos sin dinero, porque todo lo centralizan las direcciones y no se distribuye.* (...) *también se centralizó la distribución de antiparasitarios para desplazarnos*”.

En el mismo sentido, otra entrevista refirió:

“*Hay muchas cuestiones que se pueden hacer y no se hacen. Esos obstáculos no son una cuestión irresoluble. Y si pasa el tiempo y esto no se resuelve, ¿vos crees que podrías seguir trabajando ahí y amoldarte? Digamos, que eso es lo que hay*”.

Los modos en que se implementaron los lineamientos internacionales para la gestión de la hidatidosis, en este caso, reflejan matices de colonialidad a través de la influencia ejercida por los organismos internacionales en lo que se tiene que hacer, dónde y cómo. Ahora bien, quienes asumieron esta tarea y organizaron su implementación en los territorios -sin participación local en el diseño e implementación-, se enfrentaron, a su vez, con dinámicas coloniales internas desde el autoritarismo jerárquico de colegas.

En este sentido, es relevante destacar, por un lado, que las formas de globalización responden a imperativos transnacionales, en los que las condiciones locales se desintegran, se desestructuran y, finalmente, se reestructuran en forma de inclusión subalterna ^7^. Estas formas de inclusión subalterna no solo se producen desde los organismos internacionales hacia los equipos, sino que se reproducen al interior de los equipos -en base a criterios de jerarquía vinculada al género, en este caso. Se naturalizan, así, relaciones de poder donde predominan ciertas formas de conocimiento: los organismos internacionales subalternizan a las autoridades locales que, a su vez, lo hacen con los equipos locales y estos últimos, con las comunidades. Estas jerarquías se expresan en formas diferenciadas de acceso a derechos, ciudadanía, representación política y recursos económicos propios de dinámicas de colonialismo interno como componente constitutivo de la modernidad capitalista y de los Estados-nación periféricos ^32^ ^,^ ^33^.

El concepto de colonialismo interno ^32^ permite comprender que la dependencia no solo se reproduce desde “afuera” (OPS, OMS, países centrales) sino también desde adentro, en la forma en que los actores locales adoptan y naturalizan marcos foráneos sin generar alternativas situadas. Muchos actores locales, formados dentro de estas mismas lógicas, reproducen sin cuestionamiento los lineamientos internacionales, dificultando la construcción de abordajes situados. Esta naturalización de marcos exógenos limita la capacidad de imaginar alternativas que partan de las realidades locales y contribuye a mantener la dependencia estructural.

Esta experiencia ilustra cómo, incluso en intervenciones bienintencionadas, la implementación de políticas sanitarias para enfermedades desatendidas en el Sur Global puede reproducir lógicas verticales y dependencia de marcos externos, reforzando los desafíos para construir respuestas situadas y decoloniales. En síntesis, identificamos tres principales características de la gestión epidemiológica de la hidatidosis en el caso estudiado: autoritarismo jerárquico institucional, escasa articulación intersectorial y fuerte orientación hacia modelos biomédicos y tecnocráticos de intervención. Estas características reflejan una dependencia estructural de parámetros internacionales, que privilegian enfoques estandarizados, centrados en la vigilancia y el control, por sobre estrategias contextualizadas e integrales de salud pública. Estas dinámicas dependientes no solo limitan la capacidad de respuesta vinculada con las condiciones socioculturales y territoriales específicas, sino que también reproducen asimetrías en la producción de conocimiento y en la definición de prioridades sanitarias, excluyendo saberes y prácticas situadas.

### Todos somos islas: procesos de trabajo en la atención de la hidatidosis

El análisis de esta dimensión destacó dos categorías para la comprensión de las dinámicas coloniales sobre los procesos de trabajo en la atención de la hidatidosis en el caso de estudio. Por un lado, la fragmentación en la atención sanitaria y los posicionamientos individuales y, por otro, el abordaje exclusivamente biomédico y tecnocrático importado.

El análisis de fragmentación de las prácticas médicas en la asistencia contrasta con la evidencia científica sobre la necesaria integración de saberes, instituciones y actores. Esto trae aparejadas complicaciones evitables, por ejemplo, el no realizar la notificación obligatoria de la enfermedad por falta de comunicación entre profesionales intervinientes, lo cual impacta en la cantidad de medicación disponible para el tratamiento (cubierto por un programa nacional). La fragmentación se percibe como un trabajo en “islas”, vinculado al individualismo. Así lo refiere la persona entrevistada C:

“*Tenemos grandes profesionales que trabajan como islas. No hay forma de que trabajen en conjunto. Porque cuando quieres trabajar en conjunto, vas a la pelea. Y uno trata de que sea interdisciplinar. Pero aquí trabajamos como islas* (...) *porque somos individualistas, no tenemos quien nos reúna, estamos todos sobrecargados de trabajo, el poliempleo y todo se vuelve prácticamente automático* (...) *y tampoco se mide la calidad.* (...) *La gente de aquí* [los profesionales] *sabe mucho, tenemos la infraestructura, el personal capacitado, tienen ganas, pero estamos sobrepasados de trabajo. El médico que investiga no hace asistencia, porque no se puede hacer las dos cosas, y no hay equipos de gente para hacer recolección de datos, en fin* (...) *Los pacientes gastan mucho para venir, los recursos están, pero nosotros trabajamos como islas* (...) *el veterinario sabe mucho de perros, pero no de humanos y no hay una comisión veterinario-médico y si se compra un ecógrafo, pero no se compran antiparasitarios, qué sentido tiene?* (...) *bueno cuando vos vas con todas estas ideas básicas, te hacen así tá* [aplasta con la mano el escritorio] *como mosquito*”.

En el mismo sentido, otra entrevista refiere:

“*Las pautas están claras, los recursos, aunque son escasos, están. Lo que falta es el esfuerzo articulado e interinstitucional. Y que la persona pueda tener espacios fijos de detección para no andar deambulando*”.

En contraposición, la Iniciativa de Eliminación de Enfermedades ^34^ -en la que se encuentra la hidatidosis- propone adoptar un enfoque integrado, que implica movilizar a todo el sistema de salud para que los programas y servicios no operen en compartimentos estancos, sino considerando necesidades específicas de los grupos y contextualizando problemas transfronterizos y regionales vinculados a la eliminación de estas enfermedades. Asimismo, se enfatiza ofrecer una atención más centrada en la comunidad afectada que en la enfermedad.

Sin embargo, de acuerdo con las entrevistas, las prácticas de atención reflejan abordajes centrados en la enfermedad, con enfoques biomédicos y tecnocráticos, subregistro de información relevante -condiciones de vida y de producción- y sin relevamientos claros en sistemas de información, aspectos clave en su control y eliminación ^5^. Esta falta de relevamiento se atribuye a la escasa comunicación entre profesionales, que afecta la definición de responsabilidades y tareas. En ese sentido, la persona entrevistada E refirió que:

“*Detectamos esta desconexión en la provincia, incluso no solo para la prevención, el diagnóstico precoz, sino también en la trazabilidad de los casos que llegan como sospechosos y que después no se devuelve la información: si el caso sospechoso efectivamente dio positivo o no, y si lo fue, qué más van a hacer en ese contexto social y familiar.* (...) *Las dificultades que se encuentran más profundas en este asunto son la educación, la prevención y el seguimiento.* (...) *Tampoco hay un proceso de sistematización estadístico desde el Ministerio, que determine una manera de sistematizar. Hay que articular con el Sistema Educativo, hay que trabajar con la comunidad*”.

Respecto de cómo perciben a las familias respecto de las intervenciones, la persona entrevistada C refirió:

“[Se refiere a las familias a las que atienden por hidatidosis] *Muy preocupadas por la salud. Muy entregados a lo que dice el médico. Pero eso sí, les sirve el modelo paternalista. Porque es gente del interior con escasos recursos* (...)*. Ellos vienen entregados, yo les digo ‘venga con bolso’* [para quedarse]*. Y la gente cumple*”.

Los procesos de atención en hidatidosis en el caso estudiado pueden caracterizarse, entonces, de acuerdo con tres aspectos: fragmentación de servicios y de disciplinas; subregistro de datos; y abordajes biomédicos y tecnocráticos estandarizados que desatienden saberes (e intereses) locales. Esta matriz refleja huellas de colonialidad que se expresa también en la escasa participación de comunidades rurales e indígenas en la toma de decisiones sanitarias, lo que perpetúa formas de exclusión epistémica y política. En este caso, la hidatidosis es abordada con foco en la enfermedad -más bien en la detección del quiste- como una problemática individual, técnica y biomédica, desvinculada de su determinación social estructural.

### Investigación sanitaria institucionalizada: alcances y limitaciones en el abordaje de la hidatidosis con perspectiva local

El análisis de esta dimensión destaca categorías como la dependencia académica articulada con dinámicas de violencia epistémica ^35^ como forma de reproducir la subalternización. La investigación sanitaria institucionalizada puede definirse como un conjunto de actividades orientadas a producir conocimientos sobre salud-enfermedad que se desarrollan dentro de marcos formales (universidades, hospitales, organismos estatales o internacionales) y responden a normativas, agendas y prioridades establecidas por entidades públicas o privadas. En relación con otras formas de producción de conocimiento en salud (como los saberes comunitarios o las prácticas populares), la investigación institucionalizada, muchas veces, logra mayor legitimidad en los procesos de decisión, pero también suele estar más alejada de las experiencias locales y privilegiar dinámicas que reproducen asimetrías.

Desde 2016, capacitaciones de la OMS impulsaron acciones de control en escuelas de Santiago del Estero, incluyendo detección, prevención y entrega de antiparasitarios (Informe Loreto, 7 de junio y 21 de junio de 2017). Esto derivó en un proyecto de plan provincial de hidatidosis con articulación interinstitucional, cuyo avance se frustró por falta de decisión política y dinámicas jerárquicas. Los informes reflejan un enfoque centrado en lineamientos externos, con comunidades tratadas como objetos de intervención más que como actores y con escasa autocrítica sobre limitaciones y conflictos en la implementación. Como desafíos del plan provincial, se reconocía la necesidad de incorporar la participación local en el control de faenas domiciliarias y la implementación de pozos sanitarios para disminuir la transmisión de la enfermedad ^36^.

A pesar de formas importadas de trabajo, los equipos técnicos reconocen un vacío importante en torno a la escasez de la participación social en sus propuestas, algo que se advierte como desafío en los informes analizados:

“*Las estrategias de trabajo fueron las siguientes: articulación interinstitucional; vigilancia epidemiológica; control del ciclo del parásito; atención a las personas; promoción de la salud; evaluación.* (...) *Tuvo buena recepción la implementación de las capacitaciones en las escuelas, al igual que la realización del escaneo, logrando un mayor compromiso de las familias y el hospital local para el seguimiento de los casos.* (...) *Como desafíos está incorporar la participación local en el control en las faenas domiciliarias*” (Plan Provincial de Hidatidosis, 2016-2017).

Se percibe que el equipo técnico tiene conciencia de la importancia del trabajo de los equipos locales para generar vínculos con las familias y poblaciones que asisten de manera continua. Con todo, se advierte que la actuación de estos equipos locales reproduce la aspiración biomédica en el abordaje construido a partir de la intervención de los organismos internacionales. Allí, la persona entrevistada A reconoce que:

“*Es nuestra meta fundamental: los equipos de salud locales. Quizás ellos no hacen el trabajo de ir, de ver el estado de salud de la persona o explicarles nada, pero este nexo que ellos tienen, permite lograr que llegue la medicación,* (...) *de alguna manera también hace que la persona no tenga que trasladarse por el medicamento*”.

Las personas destinatarias asisten a las actividades definidas desde el equipo técnico y prestan sus cuerpos para generar datos a través de protocolos diagnósticos y terapéuticos estandarizados (como el tamizaje poblacional a partir de ecografías en escuelas). La persona entrevistada B advirtió que los modos de implementación llevados a cabo les permitieron percibir sensaciones de “invasión”:

“*Es como que la gente se siente invadida en sus costumbres, en sus hábitos, totalmente.* (...) *O creen que van a tener que pagar algo* (...) *Creen que se va a ver afectada su economía y hay que hacerles entender que no es así.* (...) *Entonces hay que aprovechar para alinear un solo mensaje para todos*”.

Estos datos permiten reflejar dinámicas de violencia epistémica ^35^ como formas de dominación en la producción, circulación y reconocimiento del conocimiento. Esta se manifiesta en la negación de la agencia epistémica de sujetos subalternizados, la explotación no reconocida de sus recursos, la desautorización académica, el extractivismo epistémico y la dependencia intelectual. En este caso, para generar datos y alinearse a las directrices de los organismos internacionales, se llevaron a cabo abordajes que fueron identificados como invasivos sin problematizarlos.

Ampliando esta perspectiva, trabajos sobre la Teoría de la Dependencia Académica en Salud en Argentina ^24^ ^,^ ^37^ evidencian que la agenda de investigación de organismos estatales en Argentina responde a prioridades globales que no siempre reflejan necesidades locales. Además, está orientada a enfermedades rentables para el desarrollo de intervenciones terapéuticas, cuyos principales actores son grandes instituciones académicas de países centrales en alianza con empresas farmacéuticas ^37^.

Desde las ciencias sociales, se ha cuestionado la narrativa tecnocrática que reduce las enfermedades a problemas de acceso a aspectos específicos, como medicamentos o diagnósticos, sin considerar las condiciones históricas, políticas y económicas que favorecen su persistencia. Se ha destacado, por ejemplo, cómo las enfermedades desatendidas condensan relaciones de colonialidad del saber y del poder, al ser producidas como “problemas” a resolver por intervenciones externas, muchas veces desvinculadas de las necesidades, saberes, derechos y prioridades de las comunidades afectadas ^7^ ^,^ ^10^ ^,^ ^38^ ^,^ ^39^. En este sentido, estas enfermedades representan un escenario privilegiado para revisar los límites de la Salud Global como propuesta político-epistémica y para fortalecer enfoques centrados en la soberanía sanitaria, la descolonización de la salud-enfermedad y la participación comunitaria en la definición de agendas y acciones.

Estas percepciones ponen en evidencia que reconocer la verdadera dimensión de la desatención implica no solo describir la carga epidemiológica, sino también cuestionar las racionalidades que orientan políticas y prácticas médicas que, pese a los avances, reproducen configuraciones de subalternidad y dependencia al no incluir a las personas destinatarias en el diseño, implementación y evaluación de las estrategias. Las características que asume la investigación de esta enfermedad desatendida en el caso de estudio revelan formas coloniales de producción de conocimiento, que refuerzan una separación entre saberes expertos y conocimientos situados, al tiempo que se invisibilizan las experiencias de las comunidades rurales e indígenas más afectadas por la enfermedad. La mirada tecnocrática protagonista se refuerza a partir de la dependencia de capacitaciones y financiamiento externo que valorizan indicadores cuantificables y descontextualizados. Esta mirada tiende a despolitizar el análisis de los datos, lo cual reduce la capacidad transformadora de la investigación en torno a problemáticas históricamente arraigadas, como la hidatidosis. En síntesis, se identifica una institucionalización de la investigación sin mecanismos de participación social y descontextualizada, que afianza violencias epistémicas y tensiona el potencial transformador de la producción científica en salud pública. 

## Consideraciones finales

El estudio analizó políticas sanitarias y prácticas médicas en torno a la hidatidosis como enfermedad desatendida en Santiago del Estero (2015-2023). Si bien la enfermedad se aborda hegemónicamente como un problema técnico, también constituye un espacio de disputa política, ética y epistemológica. Las políticas, alineadas con directrices internacionales, tienden a reificar comunidades y equipos técnicos, mientras que la atención médica presenta fragmentación, tecnocracia y prácticas autoritarias que refuerzan la desatención y la escasa articulación entre saberes expertos y comunitarios.

El trabajo presenta limitaciones vinculadas al carácter de estudio de caso y a restricciones en el acceso a actores y datos institucionales. Asimismo, se reconoce una distancia epistémica respecto de sujetos locales (trabajadores rurales, familias campesinas-indígenas, comunidades criadoras y agentes sanitarios comunitarios), cuyos saberes y estrategias no fueron incorporados. Sin embargo, estas limitaciones, lejos de invalidar los datos, convocan a profundizar líneas de investigación participativa, comparativa y situada, que integren saberes locales, experiencias comunitarias y perspectivas interculturales críticas en torno a los efectos de la Salud Global en contextos periféricos.

Avanzar hacia políticas efectivas exige ir más allá del control técnico (tratamientos farmacológicos y campañas de desparasitación), incorporando las condiciones socioeconómicas, territoriales y políticas que sostienen la endemicidad. Se identificaron como necesidades prioritarias el acceso sostenido a servicios veterinarios, campañas periódicas, diagnóstico temprano y canales de comunicación más fluidos con la población rural. Fortalecer el protagonismo comunitario implica capacitar referentes locales, incluir organizaciones productivas y actores territoriales en el diseño y seguimiento de acciones y promover dinámicas horizontales que puedan sostenerse desde los territorios.

En clave decolonial, se propone contrarrestar la violencia epistémica derivada de la adopción acrítica de protocolos externos, adaptando guías a contextos y saberes locales, así como negociar con organismos internacionales en condiciones que permitan definir prioridades desde el territorio. La formación crítica de equipos sanitarios es clave para reducir la reproducción automática de marcos foráneos y construir políticas sanitarias autónomas, participativas y culturalmente pertinentes. Estos hallazgos abren una agenda orientada a descolonizar políticas y prácticas vinculadas a enfermedades desatendidas, fortaleciendo capacidades locales y garantizando la participación real de comunidades rurales e indígenas en la toma de decisiones sanitarias.

## Data Availability

Los datos de la investigación están disponibles previa solicitud a la autora correspondiente.
